# Sensitivity Analysis of Localized Electrochemical Impedance Spectroscopy Towards Tomography-on-a-Chip

**DOI:** 10.3390/s25206393

**Published:** 2025-10-16

**Authors:** Lilia Bató, Péter Fürjes, János M. Bozorádi, Vladimir Tadić, Péter Odry, Zoltán Vizvári

**Affiliations:** 1 Institute of Technical Physics and Materials Science, HUN-REN Centre for Energy Research, H-1121 Budapest, Hungary; furjes.peter@ek.hun-ren.hu (P.F.); bozoradi.janos@ek.hun-ren.hu (J.M.B.); 2 Doctoral School on Materials Sciences and Technologies, Óbuda University, H-1034 Budapest, Hungary; 3 Department of Mechanical Engineering, Electrical Engineering and Computer Science, Technical College of Applied Sciences in Zrenjanin, 23000 Zrenjanin, Serbia; vladimir.tadic@vts-zr.edu.rs; 4 John von Neumann Faculty of Informatics, Óbuda University, H-1034 Budapest, Hungary; vizvari.zoltan@nik.uni-obuda.hu; 5 Symbolic Methods in Material Analysis and Tomography Research Group, Faculty of Engineering and Information Technology, University of Pécs, H-7624 Pécs, Hungary; podry@uniduna.hu; 6 Institute of Information Technology, University of Dunaújváros, H-2401 Dunaújváros, Hungary; 7 Bioimpedance Technologies Research Center, University Research and Innovation Center, Óbuda University, H-1034 Budapest, Hungary; 8 Multidisciplinary Medical and Engineering Cellular Bioimpedance Research Group, Szentágothai Research Centre, University of Pécs, H-7624 Pécs, Hungary

**Keywords:** electrochemical impedance spectroscopy, lab-on-a-chip, organ-on-a-chip, microfluidic systems, sensitivity analysis

## Abstract

Electrical impedance measurements are traditionally macroscopic screening techniques designed to obtain information about the macroscopic internal structure of biological systems. In order to overcome the limitations that the technology detects, mainly with the bulk properties, a miniaturization is employed by developing a complex microfluidic system to achieve cell-scale information. In this work, a microelectrode array was incorporated into a microfluidic chip, allowing localized Electrochemical Impedance Spectroscopy (EIS) measurements, providing impedance data obtained in the spatial and frequency domains simultaneously. The height of the capillary in the microfluidic system was also systematically modified; hence, three types of channels with heights of 10 μm, 30 μm, and 50 μm were developed and studied. The EIS data collection was implemented using two different strategies (two- and four-electrode techniques). Sensitivity analysis was conducted using a microbead solution, where the linear mapping of the number of microbeads along the channel was achieved by EIS. Based on the findings, a complete overview of each measurement implementation was obtained, which is well explained by the physical background presented in the paper. In the case where the capillary height (10 μm) is comparable to the diameter of the microbeads (6 μm), the four-electrode technique detected the beads in a wider frequency range (approximately between 500 Hz and 50 kHz), while the two-electrode technique detected the beads in a narrower frequency range (approximately between 30 kHz and 300 kHz) with correlation greater than 0.9. In all other cases, a medium (or weak) correlation was found between the impedance data and the longitudinal bead distribution. Based on the results, the technology is ready for further development and adaptation for cell culture purposes.

## 1. Introduction

In recent years, when organ-on-chip (OoC) technologies have emerged as the novel paradigm in personalized medicine, they have become quite significant not only in drug development, but also in cancer research and in disease modeling too, especially as the U.S. Food and Drug Administration announced this year to phase out animal testing in favor of the more effective human-relevant methods [1,2]. This is due to the inaccuracy of results obtained in animal models when applied to humans, often leading to harmful treatments owing to differences in complexity and metabolism between species [3]. To better understand the models used in these systems, the growing demand for insights into the internal structure and function of cells and tissues has prompted the development and integration of various sensors [4,5,6]. Data from the sensors can even be combined with Artificial Intelligence (AI) and machine learning methods in order to enhance the reliability and efficiency of experimental processes, reaching greater accuracy [7]. The ideal sensor for monitoring cell behavior should be label-free, non-invasive, have real-time monitoring, have prospects for continuous sampling and analysis, and allow reliable and high-quality data collection. Among the various types of sensors, optical and electrical sensors are most commonly used to meet the aforementioned conditions [8,9]. However, optical detection methods often use fluorescent dyes that can alter cell behavior and reduce their viability [10,11,12]. When studying living cells and tissues, it is essential to minimize external environmental influences and to avoid structural damage. For this reason, electrical sensing methods like Trans-Epithelial/Endothelial Electrical Resistance (TEER), Electric Cell-substrate Impedance Sensing (ECIS), or Electrochemical Impedance Spectroscopy (EIS) are often incorporated into the different systems [8,13]. TEER is a powerful technique to characterize barrier integrity, function, and permeability by measuring the electrical resistance over a tissue barrier [14]. For this reason, cells are grown on membranes, and electrodes are placed on both sides to measure the voltage drop across the barrier. In OoC models, TEER has been applied to study the blood–brain barrier [15], the lung [16], the gut [17], and the skin [18], among many others. ECIS differs from TEER in the sense that cells are grown on the surface of co-planar electrodes, and the impedance is measured at various frequencies. This technique is used for following cell growth, spreading, attachment, morphology, and viability [19,20,21]. In contrast to the aforementioned methods, a more general and detailed analysis is provided by EIS, as a wider range of frequencies is used and more versatile electrode configurations are offered [22,23]. It is a widely adopted technique that enables non-invasive, label-free, real-time evaluation of cell properties, such as their viability, structural integrity, adhesion, and cytotoxicity [24,25,26]. This technique is readily integrated into microfluidic systems owing to their many advantages, such as high sensitivity and real-time monitoring of cells while they recreate and maintain their microenvironment [27]. Another advantage of this technique is that the system under investigation can be modeled by an equivalent circuit that enables the determination of the electrical parameters, like the resistances and conductivities, of both the electrodes and the cells [28,29]. Yeast cells are preferred as model systems due to their ease of cultivation and fast reproduction rate [30]. For this reason, many EIS applications include testing the trapping and analytical functions of the designed systems with yeast cells [31]. However, aside from yeast cells, microfluidic systems can be adapted to perform impedance-based cell analysis on different cell types, such as HeLa and other cancer cells [32,33]. EIS methods have been successfully applied to determine confluency and quantify the cell number [25], to measure cell response to external stimuli [34], to continuously monitor cell behavior [22], or even to detect and characterize bacteria [35,36].

One well-known application of EIS is Electrical Impedance Tomography (EIT), which is a macroscopic method generally used in body composition analysis and targeted diagnostic procedures, with promising applications in tumour detection and cancer research [37,38]. Despite its practicality, conventional EIT is limited to supplying qualitative information on tissue-level parameters and lacks the resolution required for cellular-level analysis [23]. This limitation can be addressed by miniaturizing the system through integration with microfluidic technologies, enabling high-resolution EIT capable of in situ three-dimensional (3D) monitoring of cell growth, structural changes, and responses to therapeutic treatments [39]. In recent years, existing devices have focused on studying single cells using tomography methods, either by trapping them or using flow cytometry [40,41]. The integrated electrodes inside these systems accomplish impedance data acquisition, which is then used to form a reconstructed image of the cells. Also, with the rising popularity of AI-based data evaluation, cells can be imaged by forgoing the conventional tomographic image reconstruction methods [42]. Existing devices use image reconstruction methods to follow hemolysis dynamics [40] or to examine the heterogeneity of individual cells originating from the same tissue [41]. EIT has proven itself as a tool for early cancer detection as well [43], by real-time monitoring of tumours [44] and by successfully differentiating between benign and malignant tumour growths [45]. These new approaches based on EIS and EIT can prove to be pivotal in developing a non-invasive method for oncological diagnosis [37]. Although the aforementioned methods offer qualitative analysis, they are not suitable for quantitative analysis. In this work, we aim to move towards creating a measurement principle for quantitative analysis that could overcome the limitations of this technique. Furthermore, the most significant limitation of EIT methods at present is the inverse problem that needs to be solved during image reconstruction [46]. Dealing with and solving this extremely difficult mathematical and physical problem requires state-of-the-art methods that significantly weaken the sensitivity and resolution of EIT images [46]. In this paper, all these image reconstruction methods are avoided, and the inaccuracy of the numerical methods used is minimized.

Impedance Spectroscopy and Tomography approaches hold great potential for the detailed analysis of site-specific cellular properties, offering real-time, high-resolution 3D insights into cell structure and behavior. These advances aim to significantly enhance applications in drug discovery, personalized medicine, and diagnostics. However, they all focus on single-cell imaging, not on monitoring whole cell populations while following their state and behavior. In this paper, a compact microfluidic system is proposed that aims to maintain the linear mapping of a microfluidic channel while monitoring the particle distribution by electric measurements.

With regard to the EIS measurement methods used in this study, a unique method is applied. This technology has been introduced by the authors, highlighting the software and measurement principle at the core of the technology [47]. Moreover, it has been successfully validated on several physical models (phantoms) [48]. Subsequently, this technology was adapted for cell cultures in in vitro studies, in terms of medical and plant biology applications as well [49,50,51]. Reproducibility, one of the cornerstones of bioimpedance studies [38], has also been confirmed for this technology. Sensor fabrication, calibration and validation were conducted at different times and locations, and tests were performed on different cell types. In addition to the previously demonstrated robustness and efficiency, it was concluded that the self-developed in vitro assay provides fully consistent and reproducible results [49]. The implementation of the technology with these features in microfluidic systems is proposed.

The development of a microfluidic cell-analytical system with promising applications in biotechnology is strongly undertaken by the research group, as previously discussed. As a first step, multidisciplinary researchers, using their expertise in materials science, microtechnology, mathematics, electrical and computer engineering, and metrology, have developed the microfluidic system described in the presented study. A previous design was developed for localizing cells in vertical traps, and the variations in the number of trapped cells could be followed by EIS [52]. The current design aims to maximize the information extracted from the measured structure using EIS measurements while keeping the complexity of implementing and applying the technology as low as possible. For this purpose, a single straight capillary was designed in the microfluidic chip, with 16 electrodes longitudinally embedded at the bottom. This solution allows the systematic multiplexing of the EIS measurements to perform spatial data acquisitions, i.e., scanning the electrical impedance along the entire length of the capillary. This enables the localization of biological changes both along the longitudinal coordinate and in the frequency domain.

The aim of the research is to develop a microfluidic sensor system capable of longitudinal detection and localization of qualitative changes in cells, in addition to their quantitative properties. Obviously, the first step is to examine how sensitive the developed measurement method is to the quantitative nature of inhomogeneity. Therefore, prior to biocompatibility testing, the proof of concept investigations were performed using microbeads. In this paper, the first proof-of-concept experiments of self-developed localized EIS measurements are presented. After the fabricated microfluidic chips are filled with microbead solution, the sensitivity properties of the chips are easily examined. Knowing the longitudinal distribution of the bead numbers, the properties of the different measurement alternatives are effectively compared. In all cases, the analyses are based on the linear correlation values between the measured frequency- and location-dependent impedance magnitude and the location-dependent microbead count data. Furthermore, this provides the opportunity to empirically investigate the longitudinal and frequency domain localization capabilities of the used measurement methods. The two fundamental methods of EIS implementation, the two-electrode (terminal) and four-electrode (terminal) techniques, provided the basis for the validation experiments. Taking all this into account, the aim of the presented research is defined as follows:

1. The application of a two- or four-electrode measurement setup is to be compared, and the most effective setup is to be determined.
2. To analyze the recorded data in the standard phosphate-buffered saline (PBS) solution.
3. A discussion is to be conducted to determine which data processing method achieves the most efficient measurement procedure.
4. A description is to be provided, based on electrochemical models, of which frequency range allows detection to be maximized (in the case of the two- or four-electrode measurement technique).
5. To establish the conditions for detecting impedance inhomogeneities in chips.
6. To observe modifications resulting from the changes to the geometry of the chip.

To reach all these conclusions, three types of microfluidic chips with different channel heights (10 μm, 30 μm, and 50 μm) were prepared, with 16 platinum electrodes placed at the bottom. Each of these constructions has been designed to allow impedance measurements to be performed with any electrode configuration. Therefore, all the chips were treated exactly the same way. They were filled with standard PBS solution, and the impedance scans were performed. Then, they were filled with the prepared PBS-based microbead solution, and another data acquisition was conducted. Finally, they were refilled with the same microbead solution, and the last data recordings were taken.

This paper is structured as follows. The Section 1 is the introduction with the literature review. The materials and methods are described in the Section 2. The Section 3 presents the results, while the Section 4 gives the conclusions and future work.

## 2. Materials and Methods

### 2.1. Materials

The microfluidic system was developed on Borofloat glass substrate from Schott AG in SU-8 negative photoresist layers from Kayaku Advanced Materials, Inc. (Westborough, MA, USA). To cover the channels with Polydimethylsiloxane (PDMS), the Dow Sylgard 184 kit was used and structured by soft lithography and modified by Poly(dimethylsiloxane-b-ethylene oxide) (PDMS-b-PEO) purchased from Polysciences Europe GmbH (Eppelheim, Germany). PBS was purchased from Sigma–Aldrich Ltd. (Burlington, MA, USA). The 6 μm polystyrene pink fluorescent microbeads were purchased from Spherotech, Inc. (Lake Forest, IL, USA).

### 2.2. Sensor Fabrication

**Figure 1:**
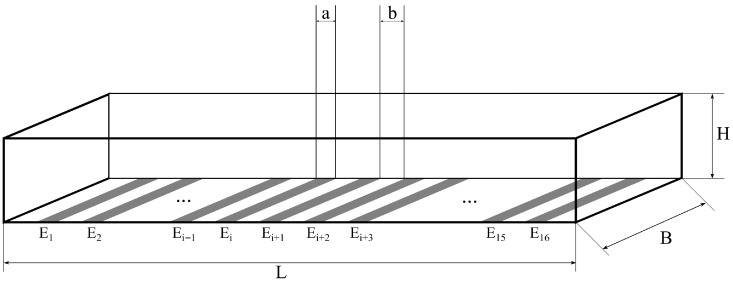
The illustration of the 16-electrode EIS capillary (the length is *L* = 2 mm, the width is B=200μm), of which 3 different heights were constructed: $H_{1}\approx 10\;\mu$m, $H_{2}\approx 30\;\mu$m and $H_{3}\approx 50\;\mu$m. The width of the electrodes is a=30μm and their distance is b=150μm for all cases.

**Figure 2:**
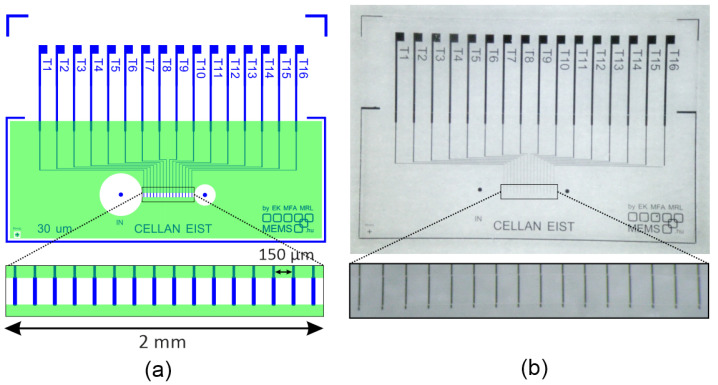
(**a**) The designed layout of the chip shows the designed electrodes in blue and the SU-8 layer in green. (**b**) The fabricated chip is shown with the platinum electrodes.

The microfluidic chip has one 2 mm long main central channel. At the bottom of the channel, 16 platinum electrodes are placed equidistantly every 150 μm. The height of the channel can be readjusted during the lithography step to fit the size of the investigated particles, which is achieved by selecting an appropriate SU-8 layer thickness. The electrode layout was designed to ensure symmetrical and equidistant architecture, making it suitable for Impedance Spectroscopy applications. The illustration of the geometry of the microfluidic channel and the embedded electrodes is shown in Figure 1, while the designed layout and the fabricated chip are shown in Figure 2.

The electrodes were patterned by lift-off lithography and were produced by vacuum evaporation of a 150 nm thick platinum layer onto a $4^{\prime \prime }$ Borofloat glass substrate (Schott AG, Mainz, Germany), using a 5 nm titanium adhesive layer. All the electrodes are 30μm wide, and the distance between the electrodes is 150μm. In order to insulate the electrodes outside the channel, the chip and the electrode surfaces are covered with a SU-8 layer. In this insulation layer, the microfluidic capillary is created during the lithography step in the negative photoresist layers. The capillary walls are developed in the SU-8 layer using photolithography on the glass substrate that incorporates the electrodes. To accomplish the exact alignment of the electrodes along the channel, a SUSS MA6 (SUSS MicroTec SE, Garching, Germany) mask aligner is used during the lithography steps.

The top roof was manufactured by standard soft lithography and replica molding in PDMS to cover the channel walls and incorporate the holes for the inlet and the outlet [53]. The master mold was developed on a $4^{\prime \prime }$ silicon wafer by photolithography using SU-8. The positive relief was cast with 1:10 PDMS containing 1:200 PDMS-b-PEO to render the PDMS surface hydrophilic. The mixture was cured at 65 °C for 2 h, and then the separate chips were cut, peeled, and cleaned in an ultrasonic shaking bath in deionized water and in isopropyl alcohol. Lastly, the PDMS was punctured at the marked places and was set onto the glass chip to enclose the capillary. To prevent leakage during filling, sufficient space is left between the edges of the chips and the holes punched for the inlets. This way, the PCB and glass chips are reusable with appropriate cleaning steps, and the PDMS top layer can either be used multiple times or changed, which enables the preliminary characterization of the system before the actual measurements.

The size of the microfluidic chip is 15×10 ${\mathrm{mm}}^{2}$. The diameter of the inlet is 500μm and the outlet is 1000μm with matching holes punched into the PDMS to fill the channels. During the measurements, three different channel heights were used: $H_{1}\approx 10\;\mu$m, $H_{2}\approx 30\;\mu$m, and $H_{3}\approx 50\;\mu$m.

**Figure 3:**
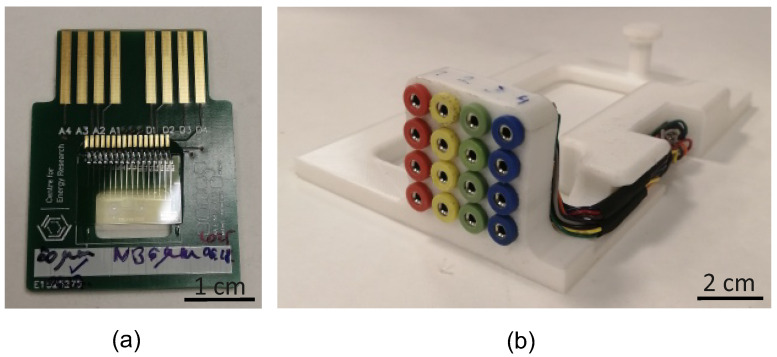
(**a**) The microfluidic chip on the PCB (**b**) and the switching matrix.

For conducting electric measurements, a specifically designed printed circuit board (PCB) and a switching matrix were designed and developed. This way, both the connection to the measurement devices and electrode selection are facilitated. In order to parallelize optical and electric measurements, a 3D printed microscope insert was produced that can hold the PCB containing the microfluidic structure. Also, the PCB has an optical window at the center that allows parallel optical monitoring alongside the impedance measurements. The PCB and the switching matrix are shown in Figure 3.

### 2.3. Microbeads Distributions and Visualisation by Optical Microscopy

**Figure 4:**
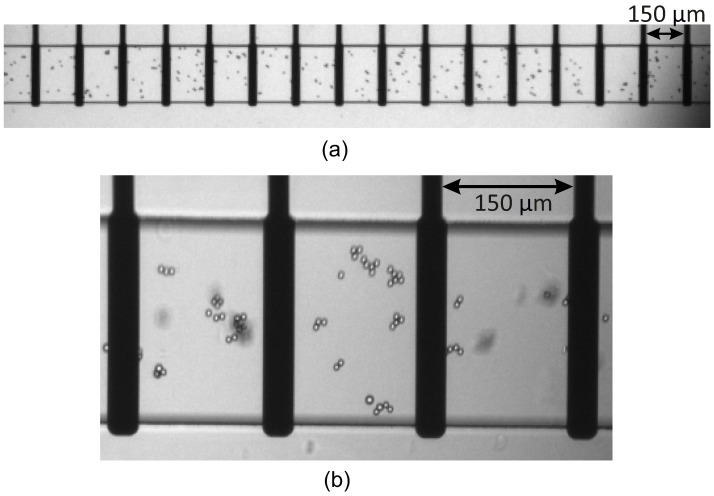
(**a**) The bead distribution along the whole channel and (**b**) an enlarged section depicting the beads between the electrodes (black).

For the experiments, a 6 μm pink fluorescent polystyrene microbeads were used. The microbead solution was made by diluting the original purchased concentration (2 mL, 1.0% *w*/*v*) to 4× with PBS. Then, the channel was filled with the solution, and the flow distributed the microbeads over the channel and between the electrodes (Figure 4).

In each case, the channel was filled with 5 μL of the diluted microbead solution. When the pressure imbalance equilibrated and the flow stopped in the channel, the final randomly generated microbead distribution was fixed by dropping paraffin oil on the inlet and outlet to seal the holes in order to prevent evaporation and further movement along the channel. Therefore, the bead distribution is kept constant inside the channel during the measurements. Images of the beads (similar to those in Figure 4) in the channels were taken prior to the electric measurements to see their distribution and to count them manually between the individual electrodes and to record their number.

The optical microscopy was performed by a Zeiss AxioVert A1 (Carl Zeiss AG, Oberkochen, Germany) inverted fluorescent microscope equipped with a Zeiss Axiocam 506 mono microscope camera. Images were taken using the Zeiss ZEN 2.6 image processing software.

### 2.4. Basics of EIS Methods

Sensitivity analysis is based on the understanding of the theoretical basis of EIS measurements, providing explanations for the phenomena involved in the frequency domain and spatial sensitivity of the methods. In order to understand the basics of EIS, it is necessary to introduce the state-of-the-art in mathematics and physics. In this approach, the material under investigation is placed in a parallel plane capacitor where the metal electrodes are placed on the two sides of the volume region. The excitation electrode is usually the electrode to which a monochrome sine wave signal is connected, while the other electrode is the ground (0 V). The distance between the electrodes is *L* and the surface area of the electrodes is *A*. Consequently, depending on the frequency range, the ions move in the direction of the oppositely charged electrodes, accumulate around them, or oscillate around their resting position due to sinusoidal excitation. The electric field generated in the measured medium is the result of the displacement of dissolved ions, which can be described by diffusion and electromigration phenomena. Another physical fact is that on the surface of the electrodes (depending on the frequency, polarity, and amplitude of the excitation), an electric double layer (EDL) is formed, whose presence and properties are essential for the theory of impedance spectra and measurement techniques [54].

The so-called Poisson–Nernst-Boltzmann model is used for mathematical modeling of these phenomena, and the linearized version of this model is usually solved for the electrochemical potential and concentration distribution in the interval $\left[-\frac{L}{2},\frac{L}{2}\right]$. The solution method assumes a solution containing monovalent ions where the dissociation of anions and cations is equal ($c_{s}$), i.e., completely dissociated. The dielectric constant of the solvent is $\varepsilon =\varepsilon_{r}\cdot \varepsilon_{0}$, where $\varepsilon_{r}$ is the relative permittivity of the solvent and $\varepsilon_{0}=8.854\cdot 10^{-12}\frac{F}{m}$ is the vacuum permittivity. The diffusion coefficients of the ions are generally assumed to be equal (*D*). The electrodes are blocking electrodes, which means that no chemical reaction takes place on their surface, and no material flows through them; hence, the charges accumulate on their surface. The electrochemical cell is also characterized by mass and charge conservation; therefore, the source of the electric field is the difference in the concentration of the ions. The impedance of the electrochemical cell is determined using Ohm’s law, using the electrochemical potential and the external current at the excitation electrode. Since the measurement is performed using a small excitation amplitude, the response of the cell to an external signal is linear. According to Barnavelli et al. [55], the impedance of the cell (Z(jω)) is expressed with the following formula:

(1) $$Z\left(j\omega \right)=R\cdot \frac{1+j\omega \tau_{RC}\sqrt{1+j\omega \tau_{D}}}{j\omega \tau_{RC}{\left(1+j\omega \tau_{D}\right)}^{3/2}},$$

where

ω is the angular frequency obtained from the frequency of the excitation signal as
  (2) ω=2π·f
*R* is the resistance of the electrochemical cell, and it can be written as
  (3) $$R=\frac{L}{A}\cdot \frac{1}{\varepsilon D\kappa^{2}},$$
$\tau_{RC}$ is the timescale that characterizes the EDL formation process, and it can be expressed as
  (4) $$\tau_{RC}=\frac{L}{2\kappa D},$$
$\tau_{D}$ is the Debye time during which the ions diffuse over a distance of the order of the Debye length, and it is given as
  (5) $$\tau_{D}=\frac{1}{\kappa^{2}D}.$$

The κ is obtained using the so-called Debye length ($\lambda_{D}$) as follows:

(6) $$\kappa^{-1}=\lambda_{D}\equiv \sqrt{\frac{\varepsilon \beta }{2e^{2}c_{s}}}$$

where

β is the product of the Boltzmann constant and room temperature;
*e* is the is the elementary charge.

The derivation of Equation (1) is ensured by the approximation $\lambda_{D}\ll L$, which is also assumed for the chip illustrated in Figure 1.

**Figure 5:**
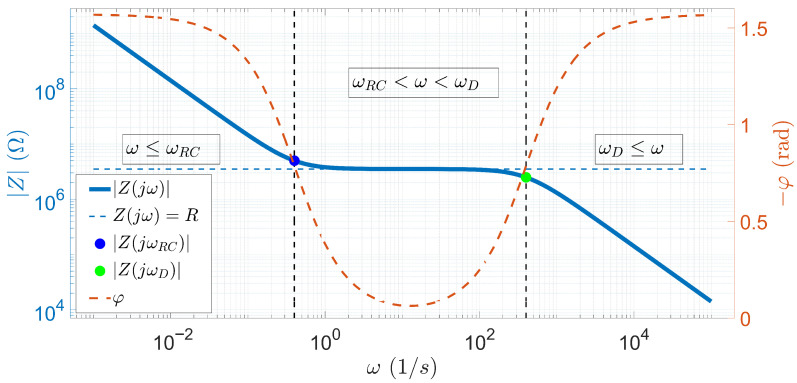
The Bode diagram of Z(jω) function calculated from the parameters *R* = 3.53 MΩ, $\tau_{RC}$ = 2.5 s, and $\tau_{D}$ = 2.5 ms using Equation (1) (the characteristic angular frequency values are $\omega_{RC}=\frac{1}{\tau_{RC}}=0.4$$\frac{1}{s}$ and $\omega_{D}=\frac{1}{\tau_{D}}=400$$\frac{1}{s}$).

The function Z(jω), defined in Equation (1), has only three parameters: *R*, $\tau_{RC}$, and $\tau_{D}$. These parameters allow us to completely describe the impedance spectrum of the material. A typical example of the function defined in Equation (1) is shown in Figure 5, where $L=10^{-3}$ m, $A=10^{-3}$ $m^{2}$, $\varepsilon_{r}=80$, $\kappa =0.2\cdot 10^{7}$$\frac{1}{m}$, and $D=10^{-10}$$\frac{m^{2}}{s}$. Considering all these values and using Equations (3)–(5), the parameters of the function Z(jω) are R=3.53 MΩ, $\tau_{RC}=2.5$ s, and $\tau_{D}=2.5$ ms, respectively. Figure 5 shows the impedance spectrum calculated with these parameters, where the left y-axis shows the magnitude of the impedance and the right y-axis shows the phase of the impedance with respect to the angular frequency.

Based on Figure 5, the fundamentals of the EIS measurements, required to understand the spectra, are obvious. As the vertical black dashed lines indicate, the spectrum is divided into three parts based on the characteristic angular frequency values $\omega_{RC}$ and $\omega_{D}$. If $\omega \leq \omega_{RC}$, the function Z(jω) is decreasing monotonically. In this range, the magnitude of the diffusion motion of ions according to Equation (4) is affected by *L*, with the distance between the electrodes. Hence, $\omega_{RC}$ is the limiting characteristic time scale of electric double layer formation, since the diffusion length is longer than $\sqrt{\frac{L\lambda_{D}}{2}}$ [55]. In contrast, in the case of $\omega_{D}\leq \omega$, because of Equation (5), the ions diffuse over a distance of the order of the Debye length, since the diffusion length is shorter than $\lambda_{D}$ [55]. In the central part, if $\omega_{RC}<\omega <\omega_{D}$, where the diffusion length is between $\sqrt{\frac{L\lambda_{D}}{2}}$ and $\lambda_{D}$, the electrochemical cell shows resistive behavior (the magnitude is assumed to be constant, |Z(jω)|=R, and the phase is close to zero). In this study, all these properties were considered in understanding the measured EIS data.

### 2.5. EIS Measurement Methods

**Figure 6:**
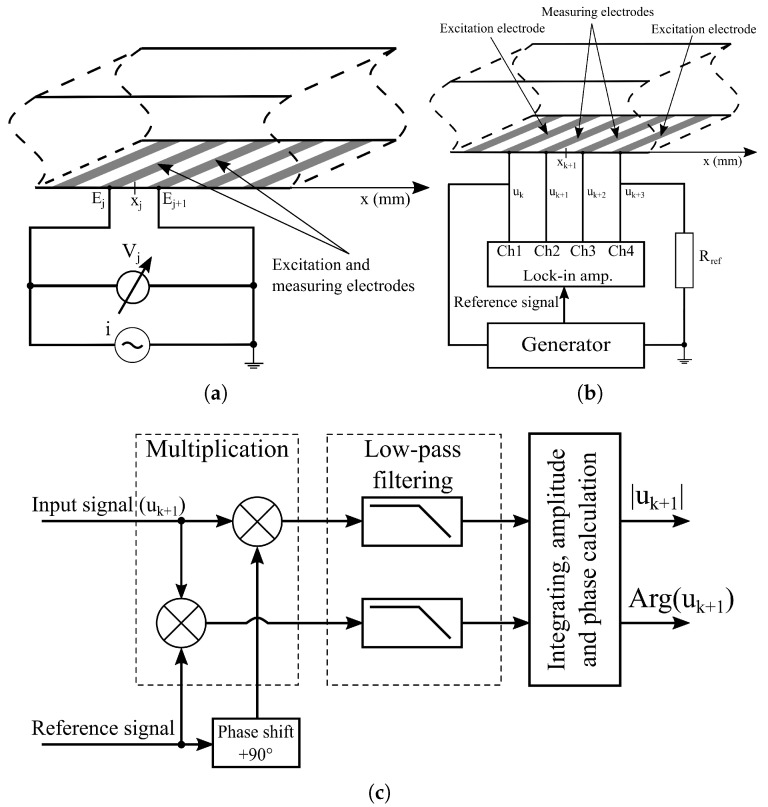
The data collection strategies and measurement principles in the case of the 16-electrode capillary illustrated in Figure 1 ((**a**) the standard two-electrode technique, (**b**) the self-developed four-electrode measurement principle based on lock-in amplifier modules, and (**c**) example of the lock-in principle implemented on different channels).

During the implementation of the EIS measurements, an electrical signal generator is connected to the excitation electrodes that generate the electric field necessary for detection (Figure 6). Since the excitation signal is a monochromatic sine wave, the resulting electrochemical processes (described in Section 2.4) create EDLs on the excitation electrodes. The detection of electrochemical potentials is achieved on the so-called measuring electrodes. The fundamental methods for implementing EIS methods are the two-electrode and four-electrode techniques [56,57]. Figure 6 illustrates the data collection strategies implemented for the 16-electrode EIS chip shown in Figure 1.

Depending on the position of the measuring electrodes, the two- or four-electrode measurement strategy is developed. In the case of the two-electrode measurement shown in Figure 6a, the voltage is measured in a very simple way on the electrodes $E_{i}$ and $E_{i+1}$, where the excitation signal has been connected. Therefore, the impedance is determined by the voltage across the investigated material and the excitation current. Naturally, the impedance spectrum is recorded by sweeping the frequency of the excitation signal. Consequently, the two-electrode method is primarily sensitive to the development and properties of the EDL. However, the sensitivity of the measurement is strongly influenced by diffusion phenomena caused by the electric field. These phenomena significantly affect the sensitivity and reproducibility of the measurement. These effects are known in the literature as contact impedances for EIS measurements. In the case of two-electrode measurements, the sum of the contact impedances and the impedance of the material is measured, making it very difficult to detect small variations in the impedance of the investigated material [58].

On the other hand, in the four-electrode measurement technique, the effect of contact impedance is eliminated by using dedicated measuring electrodes, positioned between the excitation electrodes, rather than measuring directly on the excitation electrodes (Figure 6b). This is carried out on the chip (Figure 1) using electrodes $E_{i}$ and $E_{i+3}$ as excitation electrodes, while $E_{i+1}$ and $E_{i+2}$ are used to measure the potentials as illustrated in Figure 6b. However, the technique shown in Figure 6b is not only suitable for eliminating contact impedance. This voltage comparison measurement method, developed by Vizvari et al. [47] (in addition to operating not only with the use of a current generator), provides the ability to reject the distorting effects of stray capacitances and other parasitic residual impedances in a common mode, thanks to the symmetrically designed physical implementation and the special data evaluation method. The essence of this technology is to suppress both the errors generated by the excitation electrodes and the residual impedances. This is achieved by using the reference resistor ($R_{ref}$) to shift the potentials generated in the circuit by the voltage generator with a constant value, then this constant value is eliminated in the impedance calculation as follows [47]:

(7) $$\hat{Z}\left(j\omega_{n},x_{k+1}\right)=R_{ref}\cdot \frac{u_{k+1}\left(j\omega_{n}\right)-u_{k+2}\left(j\omega_{n}\right)}{u_{k+3}\left(j\omega_{n}\right)},$$

where $\hat{Z}$ is the impedance value obtained using the four-electrode measurement method in Figure 6b at the *n*-th frequency and position $x_{k}$. In addition, the current in the loop (Figure 6b) is also measured using the reference resistor. The ratio between the digitally calculated potential differences and the measured potential on the reference resistor provides the common-mode rejection.

### 2.6. EIS Instrumentation

#### 2.6.1. Two-Electrode Measurements

**Figure 7:**
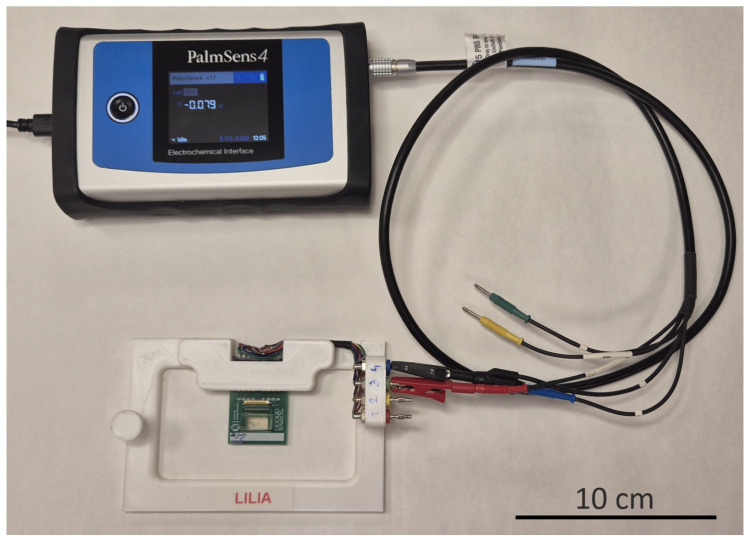
The two-electrode scanning of a microfluidic chip (the picture shows the PalmSense4 potentiostat connected to the chip).

The two-electrode measurements on the microfluidic chips were conducted using a PalmSense4 potentiostat (PalmSens B.V., Houten, The Netherlands) [59]. A two-electrode measurement set-up is presented in Figure 7.

The integrated electrode system was intended to be suitable for both two- or four-electrode measurement methods. The measurement system can sweep the frequency range between 10 μHz–1 MHz with different resolutions. The device is connected to the switching matrix by crocodile clips, and the measurements were conducted by using the associated PSTrace 5.8 software that can be used to record and evaluate the data. During the measurements, the Impedance Spectroscopy option was chosen in the measurement technique window, and the excitation voltage was set to 0.2 V. The spectra were taken between 100 Hz–1 MHz with 5 frequency points per decade.

#### 2.6.2. Four-Electrode Measurements

The four-electrode measurements on the microfluidic chips introduced in Section 2.2 were performed using a custom-designed four-channel battery-powered impedance meter instrument, which was applied by the authors in in vitro studies [47,49]. This self-developed device is a unique digital lock-in amplifier with special features, able to select the measured signal only in the close region of the user-defined reference frequency, while it very effectively blocks the other frequency components. Therefore, it is able to detect the amplitude and phase of the measured signal almost exactly at the frequency of the reference signal, but even at extremely low signal-to-noise ratios. Figure 6b,c illustrate the lock-in principle implemented in the measurement system. Figure 6b shows that the generator not only creates the electric field required for measurement across electrodes $E_{k}$ and $E_{k+3}$, but also provides a reference signal for the lock-in amplifier designed for each channel on the measuring board. The lock-in principle is implemented on the measurement board separately on the measurement channels, where Ch1, Ch2, Ch3, and Ch4 are responsible for measuring the potentials $u_{k}$, $u_{k+1}$, $u_{k+2}$, and $u_{k+3}$, respectively. Figure 6c illustrates the implementation of the lock-in principle in the case of the channel Ch2 measuring the amplitude and phase of the potential $u_{k+1}$. The realization of this principle requires three main steps: calculation of the product of the measured signal and the reference signal, application of low-pass filters, integration, and calculation of the absolute value and phase. During multiplication, the measured signal is divided into two branches and multiplied by the reference signal on one branch and by the reference signal shifted by $90^{\circ }$ on the other branch. Afterward, low-pass filters are applied to the resulting signal pairs separately. This is followed by integration, where the integration time is an integer multiple of the signal period. As a result, constant (time-independent) potential values are obtained, from which the voltage amplitude and phase are derived.

The main properties of the device are as follows:

- The excitation signal is a monochromatic sinus wave in the frequency range 1 MHz to 100 kHz, with total harmonic distortion plus noise suppression greater than 100 dB (THDN+N).
- The excitation signal is provided by a voltage generator (the maximum noise level is 1.5 $\mu V_{eff}$).
- The amplitude of the signal is manually damped between 0 and 60 dB. The default excitation (0 dB) is $1V_{p-p}$ (the excitation current is limited to 1 mA).
- The number of frequency points varies between 3 and 100 in the decades selected by the user.
- The digital lock-in algorithm uses 32-bit AD and DA converters for excitation and measurement.
- The measurement results (amplitude and phase) are calculated using IEEE 754 64-bit double precision floating-point operations [60].
- The dynamic range of the measurements is at least 160 dB.
- The accuracy (defined by the physical model, phantom [48]) is at least 1% for the resistive and at least 5% for the capacitive components.
- Battery operation with a maximum of 6.5 h.
- Currently, the main disadvantage of the technology is the offline data evaluation.

**Figure 8:**
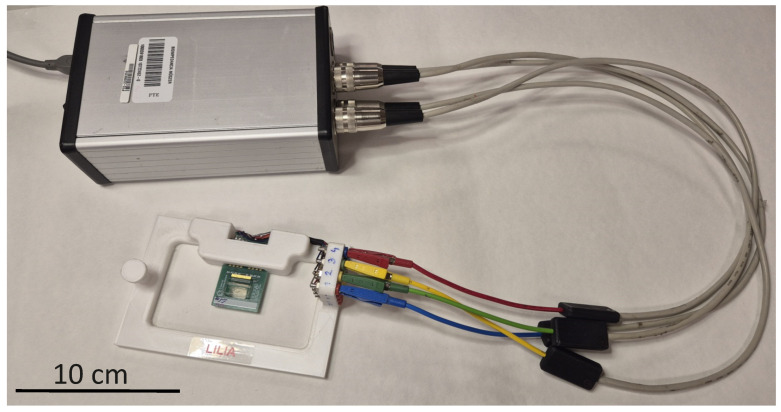
The four-electrode scanning of a microfluidic chip (the picture shows the self-developed digital lock-in amplifier connected to the chip).

In this study, the chips were scanned in the frequency range from 100 Hz to 100 kHz, with 10 frequency points per decade; hence, each data vector $\hat{Z}\left(j\omega ,x_{k}\right)$ consists of 30 elements (defined in Equation (7)), i.e., the four-electrode impedance spectrum recorded at the position $x_{k}$. The damping of the generator signal was 20 dB, which implies a sine signal of amplitude $0.1V_{p-p}$. All EIS data have been saved to csv files. The assembly using a self-developed four-electrode system is presented in Figure 8.

### 2.7. Data Collection and Evaluation Methods

The basis for the evaluation, representation, and comparison of the data with the bead counts (obtained using the methodology described in Section 2.3) is the longitudinal discretization of the chip shown in Figure 1. In the case of the length interval from 0 to 2 mm, an equidistant discretization is applied, where the elements $x_{j}$ of the vector **x**$\in R^{15}$ are the distances between the longest symmetry axis of the electrodes $E_{j}$ and $E_{j+1}$ (j=1,2,…,15 in Figure 6a). The x is also the position vector used for the two-electrode measurement, where the bead numbers and impedance values are displayed. For the four-electrode measurement, the vector $\hat{x}\in R^{13}$ is defined, whose elements ${\hat{x}}_{k+1}$ are only the distances between the longest axis of symmetry of the electrodes $E_{k+1}$ and $E_{k+2}$ if now k=1,2,…,13 in Figure 6b).

In order to discuss the methods used to collect and evaluate the impedance data sets, first let us consider the two-electrode measurements explained in Section 2.5 and Section 2.6.1. The PalmSense4 was configured to perform impedance measurements at 21 frequency points at each $x_{j}$ position; hence, the EIS scanning data of a two-electrode recording were stored in the matrix Z$\in C^{21\times 15}$. In the case of the self-developed four-electrode measurement, the EIS scanning data were stored in the $\hat{Z}\in C^{30\times 13}$ matrix, since the frequency sweep was carried out at 30 frequency points. Data ΔZ and $\Delta \hat{Z}$ indicate tables corrected using the measurement results of the PBS solution. The correction was carried out as follows:

(8) $$\Delta Z=Z-Z_{\mathrm{PBS}},$$

and

(9) $$\Delta \hat{Z}=\hat{Z}-{\hat{Z}}_{\mathrm{PBS}},$$

where $Z_{\mathrm{PBS}}$ is the two-electrode data matrix measured with PBS solution and ${\hat{Z}}_{\mathrm{PBS}}$ is the four-electrode data matrix recorded in PBS solution. The impedance data matrices are displayed in a three-dimensional coordinate system as surfaces, where the vector values x are plotted on the x-axis, the frequency on the y-axis, and the magnitude of the measured impedance values on the z-axis.

The PBS results also allow the analysis of the reproducibility of the EIS measurements. In fact, when scanning the capillary, the data are systematically measured at every position. During each position shift, the measuring device is galvanically separated from the chip due to the manual repositioning of the electrode. Consequently, during the individual measurements, the EDL on the excitation electrodes redevelops at every position, causing variance in the measured data. In addition, there can also be variations in chip manufacturing that are valuable to identify through statistical analysis. Since PBS is assumed to be a homogeneous material with frequency-dependent but not location-dependent impedance, it is reasonable to perform reproducibility testing on PBS data matrices. Moreover, in this case, the effects of damage caused by repeated use are ignored, providing a complete focus on the reproducibility of the measurement technique.

The selected metric for measuring reproducibility is the Median Absolute Deviation (MAD). However, due to the significant amplitude variation between the two- and four-electrode methods, the MAD values are given relative to the median [61]:

(10) $$\mathrm{MAD}\left(X\right)=100\cdot \frac{\mathrm{median}(|x_{i}-\mathrm{median}\left(X\right)|)}{\mathrm{median}(X)}.$$

where where $X\in R^{n}$, $x_{i}\in X$ (i=1,2,…,n) and the median(X) is the median value of the data set *X* in the classical sense. The use of the MAD value has two main advantages: it is applicable without knowledge of the distribution of the data set, and it is robust, i.e., less sensitive to the outliers and therefore more related to the dispersion around the median value [61]. For each dataset, the PBS data is visualized with both impedance surfaces and cross-sections. In this case, depending on the frequency, the impedance values are shown on the left y-axis, while the MAD values are shown on the right y-axis, calculated using the impedance values measured at the locations.

Data characterizing the distribution of microbeads in the case of two-electrode measurements were stored in the vector $n\in N_{0}^{15}$, where $n_{j}$ represents the number of microbeads between the electrodes $E_{j}$ and $E_{j+1}$ (j=1,2,…,15 in Figure 6a). In the case of the four-electrode measurement, due to the arrangement of the electrodes, the elements of the vector $\hat{n}\in N_{0}^{13}$${\hat{n}}_{k+1}$ were defined as the number of microbeads between the electrodes $E_{k}$ and $E_{k+3}$ (k=1,2,…,13 in Figure 6b).

The Pearson correlation of the impedance magnitude values along the position vector x or $\hat{x}$ and the microbead number vectors n or $\hat{n}$ along the same vectors was calculated to assess the representativeness of the impedance values. The correlation coefficient was calculated based on the following definition [62]:

(11) $$r\left(X,Y\right)=\frac{\sum_{k=1}^{n}\left(x_{i}-\bar{x}\right)\left(y_{i}-\bar{y}\right)}{\sqrt{\sum_{k=1}^{n}{\left(x_{i}-\bar{x}\right)}^{2}}\sqrt{\sum_{k=1}^{n}{\left(y_{i}-\bar{y}\right)}^{2}}}$$

where $X,Y\in R^{n}$, $x_{i},y_{i}\in X,Y$ consequently, and $\bar{x},\bar{y}$ are the arithmetic mean of the values $x_{i}$ and $y_{i}$ (i=1,2,…,n). The impedance data tables measured on each chip and the microbead numbers are compared graphically. The correlation coefficient between the longitudinal impedance profile and the microbead count is computed at each frequency point and presented as a function of frequency for each chip. Based on the obtained results, the PBS impedance profiles and the microbead measurements, as well as the distributions of the microbeads and the microbead measurements, are compared. The corrected microbead impedance data defined in Equations (8) and (9) are also compared with the microbead numbers. Moreover, the correlation between the corrected microbead impedance data defined in Equations (8) and (9) and the microbead numbers as a function of frequency is also extracted.

### 2.8. Experimental Methodology

During the experiments, attention has been paid to performing the measurement techniques in the most standardized environment in order to ensure that the observed differences are actually caused by the measurement methods. Thus, the data collection is performed by implementing two electrical impedance measurement techniques. The standard two-electrode EIS measurements were repeated systematically from the first to the sixteenth electrode on adjacent ones. The four-electrode measurements were carried out using a self-developed measurement technique that is successfully applied [47]. The technique is similar to the two-electrode technique, which is applied systematically to measure the entire electrode array. However, in this case, four electrodes were selected, rather than two, to measure at a single position. Naturally, both PBS and microbead plating were also measured using this method.

The order of experiments for each chip was standardized in order to maximize the comparability of the recorded data:

1. Filling the microfluidic chip with PBS solution;
2. Two-electrode impedance scanning for PBS solution;
3. Four-electrode impedance scanning for PBS solution;
4. Filling the chip with microbead solution;
5. Covering the chip with paraffin oil;
6. Two-electrode impedance scanning for microbead solution;
7. Four-electrode impedance scanning for microbead solution;
8. Cleaning the chip, returning to step (4), and repeating steps (5) and (6) after completion.

All these steps have obviously been implemented on all three chips involved in this study.

## 3. Results and Discussion

Three types of microfluidic chips were used for investigations ($H_{1}=10\;\mu$m, $H_{2}=30\;\mu$m, and $H_{3}=50\;\mu$m based on Figure 1) in this study.

The three chips were scanned using two-electrode measurement (described in Section 2.5) for PBS ($Z_{\mathrm{PBS}}^{\left(10\right)}$, $Z_{\mathrm{PBS}}^{\left(30\right)}$, and $Z_{\mathrm{PBS}}^{\left(50\right)}$). After adding the first microbead solution, the $Z_{1}^{\left(10\right)}$, $Z_{1}^{\left(30\right)}$ and $Z_{1}^{\left(50\right)}$ data matrices were recorded. Next, the second solution was added and the EIS data matrices ($Z_{2}^{\left(10\right)}$, $Z_{2}^{\left(30\right)}$ and $Z_{2}^{\left(50\right)}$) were recorded. Altogether, this involves the recording of 9 data matrices, i.e., 135 spectra with 2835 impedance data points only for the PalmSens4 instrument described in Section 2.6.1. The same chips were investigated with the four-electrode measurements with a PBS solution (${\hat{Z}}_{\mathrm{PBS}}^{\left(10\right)}$, ${\hat{Z}}_{\mathrm{PBS}}^{\left(30\right)}$, ${\hat{Z}}_{\mathrm{PBS}}^{\left(50\right)}$), the first (${\hat{Z}}_{1}^{\left(10\right)}$, ${\hat{Z}}_{1}^{\left(30\right)}$, ${\hat{Z}}_{1}^{\left(50\right)}$) and with the second (${\hat{Z}}_{2}^{\left(10\right)}$, ${\hat{Z}}_{2}^{\left(30\right)}$, ${\hat{Z}}_{2}^{\left(50\right)}$) microbead solutions. In this case, 9 data matrices were also recorded, but now (according to the measurement principle in Figure 6b) 117 spectra have been captured, which (according to the instrument settings described in Section 2.6.2) represent 3510 impedance measurements at all frequencies and spatial points in detail. Naturally, the PBS-corrected impedance data tables (based on Equations (8) and (9)) and correlation coefficients (based on Equation (11)) were calculated from these data.

In each case, when the chips were filled with a microbead solution, the number of beads between each electrode was determined as described in Section 2.3. From these counts, the data vectors for the bead counts ($n_{1}^{\left(10\right)}$, $n_{1}^{\left(30\right)}$ and $n_{1}^{\left(50\right)}$, and $n_{2}^{\left(10\right)}$, $n_{2}^{\left(30\right)}$ and $n_{2}^{\left(50\right)}$), applied for the analysis of the measurements of two electrodes, were derived based on the descriptions in Section 2.7. The microbead number data vectors (${\hat{n}}_{1}^{\left(10\right)}$, ${\hat{n}}_{1}^{\left(30\right)}$, ${\hat{n}}_{1}^{\left(50\right)}$, and ${\hat{n}}_{2}^{\left(10\right)}$, ${\hat{n}}_{2}^{\left(30\right)}$, ${\hat{n}}_{2}^{\left(50\right)}$), for the analysis of the four-electrode measurements, were also generated from the microscopy results as described in Section 2.3.

### 3.1. The Results in Case of the PBS Solution

**Figure 9:**
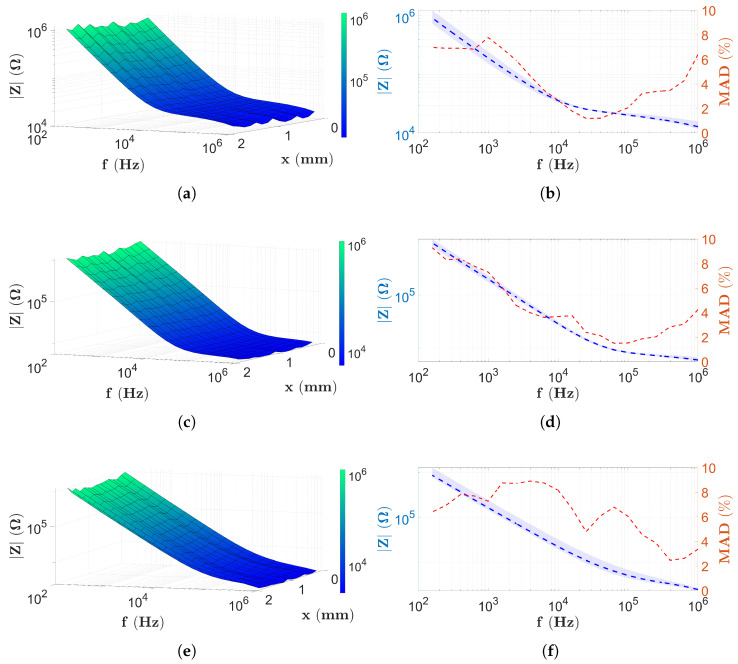
The illustration of the PBS data collected using the two-electrode measurement (Figure 6a) along the different locations and focusing only on the frequency dependence of impedance values where the red dashed line represents the median spectrum ((**a**,**b**) in a chip with height $H_{1}$, (**c**,**d**) with height $H_{2}$ and (**e**,**f**) with height $H_{3}$ (Figure 1)).

In the first case, the data for the PBS solution are presented. Figure 9 shows the EIS data measured after PBS filling (based on Section 2.8) using the two-electrode scanning method described in Section 2.5.

Figure 9a,b present the results of the scanning with two electrodes of a capillary with the height $H_{1}$. In the case of the surface shown in the figure, the frequency of the measurement is shown along the *y*-coordinate. Therefore, at each coordinate $x_{j}$, one impedance spectrum has been recorded. Thus, it can be seen that the spectra (independent of $x_{j}$) show a monotonically decreasing tendency, reaching their maximum values at low frequencies and rapidly decreasing. Approximately around 20 kHz, the rate of decrease slows drastically, and the results show a more horizontal surface (Figure 9b). This continues up to the highest frequency value of 1 MHz. The results for the chips with heights $H_{2}$ and $H_{3}$ follow the same behavior, as shown in Figure 9c, Figure 9d, Figure 9e, and Figure 9f, respectively. However, Figure 9d illustrates that the surface becomes nearly horizontal around 100 kHz. In fact, in Figure 9f, the surface gradient decreases above 200 kHz; however, even at the highest frequency values, it is not clear that the surface becomes completely horizontal. Compared to Figure 5, which illustrates the theoretical basis of EIS as described in Section 2.4, the spectra shown in Figure 9 are fully consistent with the state-of-the-art EIS theory. However, the frequency range used for the measurements no longer includes the frequency $\omega_{D}$, nor the range $\omega_{D}\leq \omega$.

The values of the high frequency range, where the impedance data are independent of both frequency and *x*-coordinate, are approximately 2 kΩ, 6 kΩ and 20 kΩ for Figure 9b, Figure 9d, and Figure 9f, respectively.

In Figure 9b,d,f, beside the cross-sections of the PBS scans, the MAD values are also shown using the red dashed lines. Using the red y-axis in Figure 9b, it is observed that between approximately 5 kHz and 700 kHz, the MAD values decrease to 5%, and between 10 kHz and 100 kHz below 2%. Figure 9d shows a similar trend. As the frequency increases, the red dotted line drops rapidly, falling below 5% above 2 kHz and below 2% above 40 kHz. These low values remain only in a narrow frequency range, since MAD values increase above 2% at frequencies over 200 kHz. A monotonic increase is seen as the frequency increases, but it does not reach 5% even at 1 MHz. The MAD data shown in Figure 9f already has a different behavior. As the frequency increases, a gentle decrease appears; values below 2% are not observed in the entire frequency range. Values below 5% only occur at the high frequencies, above 200 kHz.

**Figure 10:**
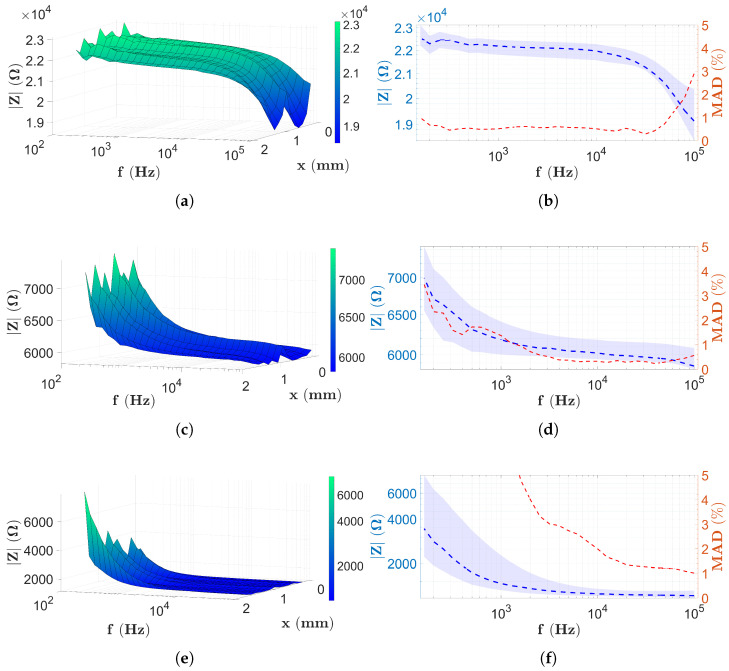
The graph of the PBS data collected using the four-electrode measurement (Figure 6b) at the different *x*-coordinates adding the frequency dependence of impedance values where the red dashed line indicates the median spectrum ((**a**,**b**) in a chip with height $H_{1}$, (**c**,**d**) with height $H_{2}$ and (**e**,**f**) with height $H_{3}$ (Figure 1)).

The two-electrode results measured on PBS are followed by the four-electrode data obtained based on Section 2.5. Figure 10 shows the results of the four-electrode scans of the chips, captured using the settings applied in Section 2.6.2.

Figure 10a,b present the impedance data for capillary height $H_{1}$. It is observed that again there is minimal change in the *x*-coordinate; hence, the findings are more related to the frequency dependence. The impedance surface now assumes a constant value at lower frequencies and only rapidly decreases at high frequencies >50 kHz (Figure 10b). In contrast, in the case of the higher capillaries (Figure 10c,d,f), a sharp decrease is observed at low frequencies, since the maximum of the spectra is measured at the lowest frequency values. However, in both cases, as the frequency increases, the impedance values remain constant (for Figure 10d at the frequencies >2 kHz and for Figure 10f at the frequencies higher than approximately 1 kHz), and this remains the same throughout the frequency range. Compared to the theoretical spectrum, it can be seen that for a capillary height $H_{1}$, the $\omega_{RC}<\omega <\omega_{D}$ and $\omega_{D}\leq \omega$ ranges coincide with the measurement frequency range. Based on theoretical results, the observed drop in Figure 5 corresponds to the characteristic frequency $\omega_{D}$. In the case of $H_{2}$ and $H_{3}$ capillaries, the situation is the same as for the two-electrode measurement. The measurement frequency range includes the impedance ranges $\omega \leq \omega_{RC}$ and $\omega_{RC}<\omega <\omega_{D}$. Thus, the change in gradient seen at low frequency corresponds to $\omega_{RC}$ according to the theory presented in Section 2.4.

The constant impedance values shown in Figure 10b,d,f for the four-electrode measurement are approximately 22 kΩ, 6 kΩ, and 1.5 kΩ, respectively.

The frequency dependence of the MAD values calculated for the four-electrode PBS scans can be observed in Figure 10b,d,f. In Figure 10b, the MAD values marked with a red dashed line remain below 1% across almost the entire frequency range; however, following a rapid increase visible from 50 kHz, they do not reach 5% even at high frequencies. Figure 10d shows a different characteristic. At low frequencies, the red curve shows a decreasing trend, and above 2 kHz, the MAD data remain below 1% across the entire frequency range. However, in the case of a capillary with a height of $H_{3}$, MAD values below 5% are only observed above approximately 1.5 kHz, reaching 1% at a frequency of 100 kHz with a monotonic decrease.

Following the analysis of the PBS data, the results of the microbead-filled chips are obtained using correlation calculations of the impedance values and the distribution of the microbead counts, as described in Section 2.7.

### 3.2. Results with Two-Electrode Measurements

**Figure 11:**
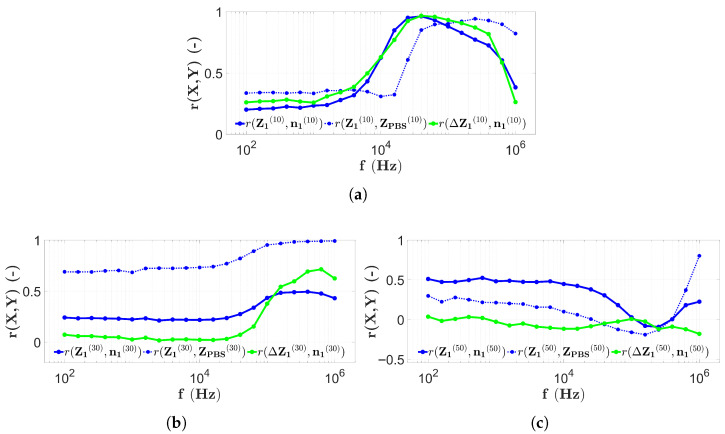
The correlation coefficients (defined in Section 2.7) calculated by comparing the distribution of the microbeads (in the first case) and the two-electrode data (Figure 6a) measured for microbeads and PBS (in the case of (**a**) $H_{1}$, (**b**) $H_{2}$ and (**c**) $H_{3}$).

The results of two-electrode measurements during the first microbead filling are shown in Figure 11.

Figure 11 is prepared as described in Section 2.7 to allow easier comparison with other cases. The frequency dependence of the correlation calculated from the longitudinal distribution of the impedance and the bead count ($r(Z_{1}^{\left(10\right)},n_{1}^{\left(10\right)})$), indicated by the blue solid line, is clearly shown in Figure 11a for height $H_{1}=10\;\mu$m. These correlation values remain constantly below 0.6 at low frequencies (<10 kHz), then after a large increase, values above 0.8 are obtained until approximately 300 kHz. Then again, the curve decreases rapidly. However, the correlation values $r(Z_{1}^{\left(10\right)},Z_{PBS}^{\left(10\right)})$ (blue dashed line in Figure 11a), comparing the impedance distributions measured in the microbead solution and the PBS, remain below 0.6 to about 11 kHz and, after a sharp increase, remain above 0.8 for the entire frequency range. The correlation between the PBS corrected impedance and the microbead number distribution ($r(\Delta Z_{1}^{\left(10\right)},n_{1}^{\left(10\right)})$), indicated by the solid green line in Figure 11a, also takes values below 0.6 at low frequencies (<10 kHz) and then increases to above 0.8 up to about 500 kHz.

The results for the correlation values as a function of frequency for a capillary with height $H_{2}=30\;\mu$m are shown in Figure 11b. However, in this case, the curve $r(Z_{1}^{\left(30\right)},n_{1}^{\left(30\right)})$ remains below 0.6 throughout the measurement frequency range, since above 100 kHz, it is above 0.5, and at all other frequencies, only low correlation values are obtained. The curve $r(Z_{1}^{\left(30\right)},Z_{PBS}^{\left(30\right)})$, derived from the comparison with the PBS results, now takes higher values, above 0.6 over the entire frequency range. Furthermore, values above 0.8 are found above 30 kHz. The correlation values from the PBS corrected impedance distribution $r(\Delta Z_{1}^{\left(30\right)},n_{1}^{\left(30\right)})$ are also low, below 0.2 up to about 100 kHz. However, at higher frequencies, there is an increasing trend, even though the maximum value remains below 0.8.

The frequency dependence of the correlation values of the data measured using the two-electrode technique in the chip of height $H_{3}=50\;\mu$m is illustrated in Figure 11c. The correlation values $r(Z_{1}^{\left(50\right)},n_{1}^{\left(50\right)})$ (indicated with a solid blue line) are above 0.4 to about 10 kHz; however, after a drastic decrease at higher frequencies, they reach a minimum (around 0) and start to increase again until the end of the frequency range. The correlation values $r(Z_{1}^{\left(50\right)},Z_{PBS}^{\left(50\right)})$ (marked with the blue dashed line) are now also very low (0 to 0.3) and decrease monotonically in the range 100 Hz–200 kHz. However, at higher frequencies, they increase very strongly and reach the maximum value at 1 MHz. Nevertheless, the correlation $r(\Delta Z_{1}^{\left(50\right)},n_{1}^{\left(50\right)})$, calculated from the PBS-corrected impedance values, is now extremely low, around 0, over the entire measurement frequency range.

**Figure 12:**
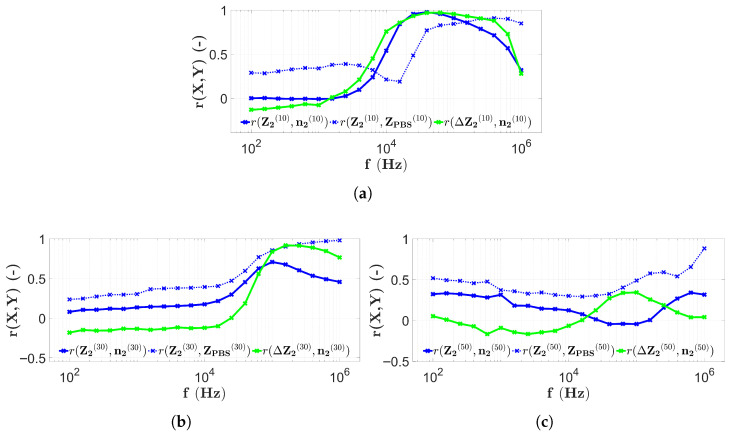
The correlation coefficients (defined in Section 2.7) calculated by comparing the distribution of the microbeads (in the second case) and the two-electrode data (Figure 6a) measured for microbeads and PBS (in the case of (**a**) $H_{1}$, (**b**) $H_{2}$, and (**c**) $H_{3}$).

In order to reproduce the previously detailed experimental results, as described in Section 2.8, the results obtained by refilling the three chips and using the two-electrode technique (introduced in Section 2.5) are illustrated in Figure 12.

The correlation values obtained after repeated microbead filling and scanning with the two-electrode technique (Section 2.8) in the capillary at $H_{1}$ are very similar to the case observed in Figure 11a. The correlation values $r(Z_{2}^{\left(10\right)},n_{2}^{\left(10\right)})$ (solid blue line in Figure 12a) are still larger than 0.8 between 20 kHz and 300 kHz, while in all other frequency ranges there are lower values. The correlation between the impedance distributions measured with PBS and the microbeads ($r(Z_{2}^{\left(10\right)},Z_{PBS}^{\left(10\right)})$, blue dashed line in Figure 11a) in this case remains higher than 0.8 at frequencies above approximately 30 kHz, while smaller frequencies show correlations below 0.4. The PBS-corrected correlation values $r(\Delta Z_{2}^{\left(10\right)},n_{2}^{\left(10\right)})$, shown by the green line, are larger than 0.8 between 10 kHz and 600 kHz, while the correlation values outside this frequency range are significantly lower.

Based on Figure 12b, the results of the microbead experiment repeated in the chip with height $H_{2}$ are analyzed. The $r(Z_{2}^{\left(30\right)},n_{2}^{\left(30\right)})$ correlation values remain below 0.8 over the entire frequency range, increasing from about 0.1 to 0.7 around 100 kHz, then decrease again. In contrast, the correlation values $r(Z_{2}^{\left(30\right)},Z_{PBS}^{\left(30\right)})$ increase monotonically at low frequencies from around 0.2 up to 1, with an increase at around 100 kHz, where all correlation values at higher frequencies are above 0.8. The PBS-corrected correlation values $r(\Delta Z_{2}^{\left(30\right)},n_{1}^{\left(30\right)})$ increase monotonically starting at −0.2 and reach high values (around 0.8 or higher) from 100 kHz.

The last group of the two-electrode measurement results is a repeat of the microbead impedance scan on the chip with height $H_{3}$. The results are illustrated in Figure 12c. The correlation values $r(Z_{2}^{\left(50\right)},n_{2}^{\left(50\right)})$ (indicated by the solid blue line) show a decreasing tendency from 0.3 down to 0, which is reached at approximately 50 kHz. Thereafter, an increase is noticed to about 0.3. On the other hand, the correlation values $r(Z_{2}^{\left(50\right)},Z_{PBS}^{\left(50\right)})$ are higher over the whole measurement frequency range, since, although there is a decreasing tendency, the correlation increases from frequency 20 kHz to 0.6 and higher. The PBS-corrected $r(\Delta Z_{1}^{\left(50\right)},n_{1}^{\left(50\right)})$ curve (indicated with the green line) also fluctuates around 0 in this case, except in the frequency range between 20 kHz and 600 kHz, where it reaches its maximum at correlation value 0.3 as well.

### 3.3. Results with Four-Electrode Measurements

**Figure 13:**
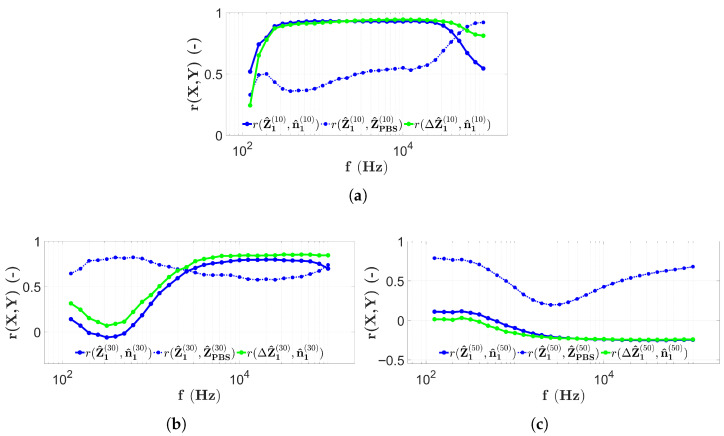
The correlation coefficients (defined in Section 2.7) calculated by comparing the distribution of the microbeads (in the first case) and the four-electrode data (Figure 6b) measured for microbeads and PBS (in the case of (**a**) $H_{1}$, (**b**) $H_{2}$ and (**c**) $H_{3}$).

The frequency dependence of the correlation coefficients calculated based on Section 2.7 and Section 2.8 is shown in Figure 13, where the results of four-electrode scanning of the first microbead filling of the chips are used.

Figure 13a shows the correlations calculated from the results of the first data collection of microbead impedance using the $H_{1}$ chip of four electrons. The correlation values $r({\hat{Z}}_{1}^{\left(10\right)},{\hat{n}}_{1}^{\left(10\right)})$ (indicated by the blue solid line) remain around 0.9 for almost the entire measurement frequency range. The only exceptions are the ranges less than 300 Hz and higher than 30 kHz, where lower correlation values are seen. The correlation between the microbead data and the PBS data in Figure 13a ($r({\hat{Z}}_{1}^{\left(10\right)},{\hat{Z}}_{PBS}^{\left(10\right)})$) remains below 0.6 for almost the entire frequency range, except for the range higher than 30 kHz, where it increases significantly to around 0.9. The PBS-corrected correlation values $r(\Delta {\hat{Z}}_{1}^{\left(10\right)},{\hat{n}}_{1}^{\left(10\right)})$ (shown with the green solid line) also take values around 0.9 for almost the entire frequency range, with the exception of the range under 300 Hz and the range higher than 30 kHz. However, for frequencies greater than 30 kHz, the correlation is still over 0.8.

Figure 13b illustrates the correlation values obtained from data measured in the chip with height $H_{2}=30\;\mu$m using the four-electrode technique. The solid blue line ($r({\hat{Z}}_{1}^{\left(30\right)},{\hat{n}}_{1}^{\left(30\right)})$) now at low frequencies shows values starting around 0, then after a rapid increase, around 2 kHz, it reaches 0.6 and oscillates around 0.8 from 8 kHz to about 80 kHz. The blue dashed line ($r({\hat{Z}}_{1}^{\left(30\right)},{\hat{Z}}_{PBS}^{\left(30\right)})$) now varies throughout the frequency range over 0.6. Between 100Hz and 3 kHz, it reaches its highest values and has its maximum segment, with values above 0.8. Between approximately 3 kHz and 30 kHz, the mentioned correlation values are the lowest, varying around 0.6, and then increase at frequencies higher than 30 kHz. The shape of the curve for the correlation values $r(\Delta {\hat{Z}}_{1}^{\left(30\right)},{\hat{n}}_{1}^{\left(30\right)})$ is exactly the same as for $r({\hat{Z}}_{1}^{\left(30\right)},{\hat{n}}_{1}^{\left(30\right)})$, with the possible difference that the $r(\Delta {\hat{Z}}_{1}^{\left(30\right)},{\hat{n}}_{1}^{\left(30\right)})$ values are higher, i.e., between the values 3 Hz and 30 kHz rather than 0.8, which remains true for frequencies above 30 kHz.

Based on Figure 13c, the curves $r({\hat{Z}}_{1}^{\left(50\right)},{\hat{n}}_{1}^{\left(50\right)})$ and $r(\Delta {\hat{Z}}_{1}^{\left(50\right)},{\hat{n}}_{1}^{\left(50\right)})$ are explained simultaneously. In fact, both lines uniformly vary around −0.25 over approximately 2 kHz. In the frequency range under 2 kHz they separate and $r({\hat{Z}}_{1}^{\left(50\right)},{\hat{n}}_{1}^{\left(50\right)})$ varies above 0, while $r(\Delta {\hat{Z}}_{1}^{\left(50\right)},{\hat{n}}_{1}^{\left(50\right)})$ fluctuates around 0. The correlation values $r({\hat{Z}}_{1}^{\left(50\right)},{\hat{Z}}_{PBS}^{\left(50\right)})$ (indicated by the blue dashed line) now take higher values, in contrast to the previous ones. For frequencies below 800 Hz and above 20 kHz, the correlation values are higher than 0.5, while between these frequencies, the values are lower. In this frequency range, the correlation decreases to approximately 0.2.

**Figure 14:**
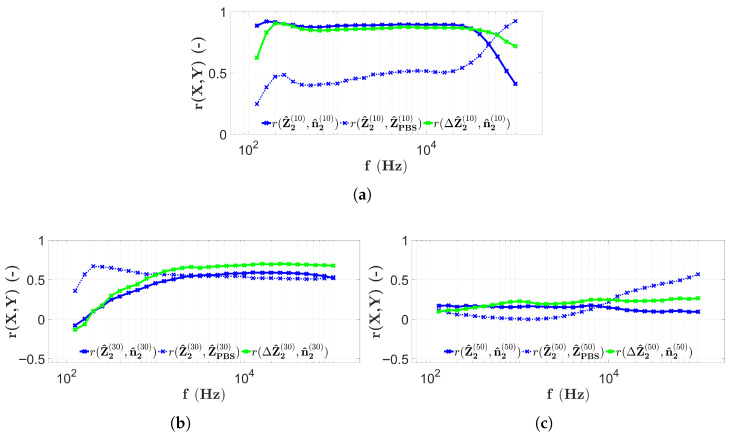
The correlation coefficients (defined in Section 2.7) calculated by comparing the distribution of the microbeads (in the second case) and the four-electrode data (Figure 6b) measured for microbeads and PBS (in the case of (**a**) $H_{1}$, (**b**) $H_{2}$ and (**c**) $H_{3}$).

The correlation values calculated from the four-electrode measurement results of the second microbead filling of the same chips are shown in Figure 14.

Figure 14a illustrates the results of repeated filling and measurements on the chip with height $H_{1}$. The correlation values $r({\hat{Z}}_{2}^{\left(10\right)},{\hat{n}}_{2}^{\left(10\right)})$ now also vary in a wide frequency range between 100 Hz and 20 kHz around 0.9. However, a rapid decrease is observed for frequencies higher than 20 kHz. The correlation values $r({\hat{Z}}_{2}^{\left(10\right)},{\hat{Z}}_{PBS}^{\left(10\right)})$, indicated by the blue dashed line, remain under 0.6 for almost the entire measurement frequency range, reaching around 0.9 after a large increase above approximately 30 kHz. The correlation values $r(\Delta {\hat{Z}}_{2}^{\left(10\right)},{\hat{n}}_{2}^{\left(10\right)})$ in this case also vary around 0.9 in a wide range between 200 Hz and 50 kHz. In the frequency ranges outside this interval, lower correlation values are observed.

Figure 14 shows the measurement results of repeated filling of chips with height $H_{2}$. The correlation values $r({\hat{Z}}_{2}^{\left(30\right)},{\hat{n}}_{2}^{\left(30\right)})$, indicated by the blue solid line, show a monotonic increase as a function of frequency from around 0 up to around 0.5 and to slightly higher, in the frequency range 1 kHz and above. The PBS-corrected correlation values $r(\Delta {\hat{Z}}_{2}^{\left(30\right)},{\hat{n}}_{2}^{\left(30\right)})$ are parallel to the previously analyzed ones, but the green solid line in Figure 14b, at frequencies higher than 1 kHz, is consistently greater than 0.6. The correlation values $r({\hat{Z}}_{2}^{\left(30\right)},{\hat{Z}}_{PBS}^{\left(30\right)})$ denoted by the blue dashed line reach their maximum values at low frequencies (between approximately 100 Hz and 1 kHz) and remain in the proximity of 0.5 at frequencies higher than 1 kHz.

The results shown in Figure 14c are derived from four-electrode measurements arising from repeated filling of the chip with height $H_{3}=50\;\mu$m. The correlation values $r({\hat{Z}}_{2}^{\left(50\right)},{\hat{n}}_{2}^{\left(50\right)})$ and $r(\Delta {\hat{Z}}_{2}^{\left(50\right)},{\hat{n}}_{2}^{\left(50\right)})$, indicated by the solid blue and green lines, respectively, also overlap in this case at frequencies lower than 2 kHz. At higher frequencies, they separate, and the blue curve varies around 0.2, while the green curve changes around 0.3. The correlation $r({\hat{Z}}_{2}^{\left(50\right)},{\hat{Z}}_{PBS}^{\left(50\right)})$ (between PBS and microbead results) also takes higher values (>0.4) in the frequency ranges higher than 20 kHz. The maximum (around 0.6) is also seen at 100 kHz. However, in the whole measurement frequency range, low correlation values are found with a minimum of approximately 0.

After a detailed analysis of the findings, in the following subsection, the connections between the individual results are examined and discussed in detail.

### 3.4. Discussion

In this study, the self-developed microfluidic chips with 16 electrodes and capillaries with different heights ($H_{1}$, $H_{2}$, and $H_{3}$) (Section 2.1) were analyzed. The investigations, using two different alternative implementations of EIS, were performed firstly with PBS and then with solutions containing microbeads (Section 2.8). All the results of the PBS measurements (Figure 9 and Figure 10) suggest that the variation in the spectra along the *x*-coordinate can be neglected, the spectral distributions for PBS are longitudinally homogeneous, and the error due to the geometric inaccuracies of the chips is not significant. In the case of the impedance data of the two-electrode technique, the low-frequency behavior of the spectra is identical, i.e., starting from MΩ magnitude, they decrease monotonically according to the theoretical results described in Section 2.4 and presented in Figure 5. The monotonically decreasing region is typically followed by a constant surface area regardless of the electrode height. A shift of the characteristic frequency, denoted by $\omega_{RC}$ in the theoretical principles (Section 2.4), is also observed for the monotonically decreasing and constant surfaces. Also, as the height of the chip increases, $\omega_{RC}$ shifts to the higher frequencies. It is difficult to determine whether the constant surface is displayed in the measurement frequency range for the chip with height $H_{3}=50\;\mu$m. Using the four-electrode measurement technique, in Figure 10, it is interesting to note that the horizontal plane range is wider than with the two-electrode technique. Moreover, only in the case of the chip with height $H_{1}$, the characteristic frequency, denoted in theory by $\omega_{D}$, was detected. For the measurement of higher capillaries, $\omega_{D}$ is already shifted out from the measurement frequency range (similar to the two-electrode technique), although the cut-off associated with a characteristic frequency similar to $\omega_{RC}$ appears in the measurement frequency range. Regardless of this, most of the spectra also appear in a horizontal plane. The PBS data can also be used to estimate the reproducibility of EIS measurements by calculating the MAD values along the longitudinal coordinate (Equation (10)). In the case of two-electrode measurements, MAD values are generally below 5%. It is interesting to note that at low frequencies, MAD values are greater than 5% regardless of the capillary height. The best results (<2%) are obtained for capillaries with a height of $H_{1}$ in a narrow range between 10 kHz and 100 kHz (Figure 9b). The MAD values for the four-electrode measurements with capillaries of heights $H_{1}$ and $H_{2}$ are significantly lower than in the case of two-electrode measurements, since the typical values remain below 1% (Figure 10b,d). In contrast, MAD values are significantly higher for the chips with height $H_{2}$ (<2 kHz) and $H_{3}$; however, they generally remained below 5%. In the case of the four-electrode measurements, the maximal MAD values are calculated for the chip with a height of $H_{3}$ (<1.5 kHz in Figure 10f).

**Figure 15:**
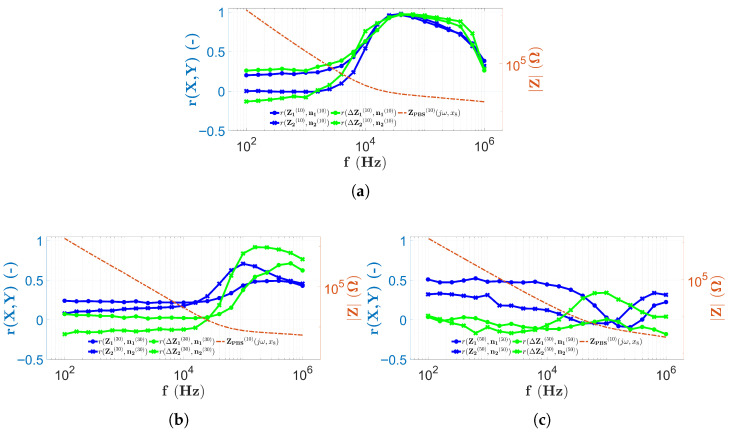
Comparison of the correlation values with a typical impedance spectrum (indicated with a red dashed line) measured on PBS solution using the $E_{8}$ and $E_{9}$ electrode pair in two-electrode technique (in the case of (**a**) $H_{1}$, (**b**) $H_{2}$ and (**c**) $H_{3}$).

To summarize the measurement properties of the EIS scans, the results of the two-electrode measurements are shown in Figure 15 by combining them with a typical spectrum recorded during PBS data collection using the appropriate technique at the center of the chip measured at electrodes $E_{8}$ and $E_{9}$.

In Figure 15a, all results obtained using two-electrode scanning are shown in a chip with height $H_{1}$. From Figure 15a, it is obvious that the correlation values remain low (<0.5) until the PBS spectrum (indicated by the red dashed line) takes on high impedance values. As the slope of the red curve decreases and enters the near horizontal range, the correlation values also increase significantly. Another interesting feature is that above 500 kHz, the correlation values marked by the solid line decrease, while the correlation values between PBS and microbead counts remain high (Figure 11a and Figure 12a). The previous findings are also true for the $H_{2}$ (Figure 15b) and $H_{3}$ (Figure 15c) chips, with the difference that, as observed from the PBS spectrum, the $\omega_{RC}$ frequency is shifted to the higher frequencies. The correlation values follow a decreasing trend as a function of capillary height. In the case of the chip with height $H_{1}$, each of the solid lines shows a strong correlation in the higher frequency range. For the height $H_{2}$, the situation is not evident since, in contrast to the blue solid lines, the PBS-corrected results show a stronger correlation. For the case of the height $H_{3}$, no correlation is found between the microbead counts and the impedance data in any frequency range.

**Figure 16:**
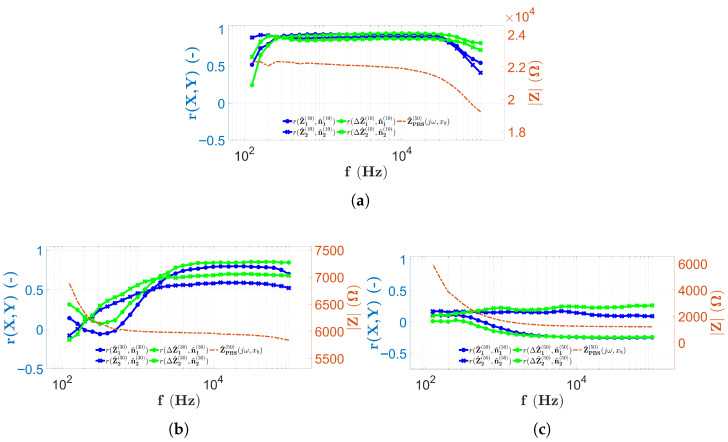
Comparison of the correlation values with a typical impedance spectrum (indicated with a red dashed line) measured on PBS solution using the $E_{8}$, $E_{9}$, $E_{10}$ and $E_{11}$ electrodes in four-electrode technique (in the case of (**a**) $H_{1}$, (**b**) $H_{2}$, and (**c**) $H_{3}$).

A summary of the results of the four-electrode EIS scans is shown in Figure 16. Similarly to the previous results, a typical PBS spectrum is also given, measured at the center of the capillary at electrodes $E_{8}$, $E_{9}$, $E_{10}$, and $E_{11}$.

In the case of four-electrode scans, interesting findings are possible. The results shown in Figure 16a show that the correlation values are also high in the frequency range where the PBS impedance spectrum assumes a constant value. This is also observed in Figure 16b. Notably, for both heights, when the spectrum indicated by the dashed red line changes from a constant value, the correlation values fall drastically (Figure 16a at high frequencies and Figure 16b at low frequencies). The decrease in the maximum correlation values with capillary height is also observed in this case, but for the $H_{2}$ chip, a stronger correlation is observed as a consequence of the PBS correction. The chip with the height of $H_{3}$ is an exception, although the appearance of the characteristic frequency $\omega_{RC}$ is clearly observed, its effect is not visible in the correlation values, since they remain low throughout the whole measurement frequency range. In the case of the four-electrode measurements, it is also interesting to note that the correlation of the microbead compared to PBS impedance measurements increases with the increase in height. The only observed case where a strong correlation was measured in the microbead counts and a weak correlation in the PBS impedance data is for the chip with height $H_{1}$ (Figure 13a and Figure 14a).

## 4. Conclusions and Future Works

In this paper, a self-developed microfluidic device based on lab-on-a-chip technology is introduced, in which a 16-electrode array is integrated. Localized EIS measurements have been performed in the case of a capillary integrated in the microfluidic sensor by implementing two-electrode and a special four-electrode technology. A complete sensitivity analysis was performed using PBS and microbead solutions. The robustness and efficiency of each EIS measurement technique were compared using correlation calculations of the longitudinal bead counts and measured impedance values. The understanding of the measurement features was explained using the PBS results and the state-of-the-art EIS theory. Conclusions from the results were drawn from a large amount of impedance data.

During the completion of this study, a data structure was developed to store the results of an EIS scan in a data matrix. For the first time, the EIS data matrices of the PBS solution were recorded for chips with heights $H_{1}$, $H_{2}$, $H_{3}$ for both measurement implementations. In general, the observation indicates that in the two-electrode measurement frequency range (100 Hz–1 MHz), only a relatively narrow interval of the resistive behavior of the cell can be detected. In contrast, for the four-electrode measurement, a much wider resistive plateau was detected, covering almost the whole frequency range (100 Hz–100 kHz). Moreover, it was found that with increasing capillary height, the characteristic frequencies defined in the theoretical basis were shifted towards higher values. The MAD values also indicate that the reproducibility of EIS measurements is the highest in the resistive range. Since four-electrode measurements tend to focus on this resistive range in the spectra, this measurement technique generally provides better reproducibility.

In the case of microbeads, the sensitivity of the impedance data matrices has been fully analyzed. The correlation of the EIS data matrices measured on microbead solutions with the longitudinal microbead counts and separately with the chip geometry (through PBS results) was examined. Note that the microbead distribution is randomly developed in the microfluidic channel. Moreover, impedance data were corrected using the data measured in the pure PBS media in an effort to increase the correlation. Summarizing the microbead results, it can be clearly concluded that in microfluidic systems, it is only possible to detect microbeads with the appropriate sensitivity, i.e., with high correlation, if the measurement is performed in a frequency range where the chip exhibits resistive behavior. Consequently, the four-electrode system is the most suitable for this purpose, where the capillary height is comparable to the diameter of the bead. At frequencies lower than the range typical for resistive behavior (<$\tau_{RC}$), the diffusion effects caused by the measurement are dominant; therefore, the detection of microbeads was impossible in all cases. It is an interesting observation, since in the frequency range above the resistive plateau (>$\tau_{D}$), the EIS data correlate more with the capillary geometry (i.e., PBS data) rather than with a capacitive sensor. In addition, it was demonstrated that increasing capillary height reduces the sensitivity of EIS methods to microbeads. Therefore, the only limitation of the studied measurement technique is that, in practice, the capillary needs to be adjusted to the size range of the cells to be examined.

The findings obtained in the present study allow us to estimate the sensor geometry, the measurement approach and, through these, the sensitivity and effectiveness of the sensor depending on the size of the cell type to be monitored. The most important step after this is considered to be the thorough validation of the biocompatibility of various microfluidic chips optimized for different tasks, as well as the feasibility of culturing cells within them. These results are essential for the design and implementation of future biological experiments. Naturally, following the successful completion of all these objectives, the research group intends to use and further develop the complete technology for various OoC applications in the form of target prototypes. The results and experiences gained during this study are also applicable to biological studies. The electrical behavior of microbeads is very similar to the low-frequency behavior of cells. In this case, the cell membrane acts as an insulator, and the electric field is developed only in the extracellular space, as is the case with beads. Therefore, due to the existence of correlation, the measurement methods presented in the paper (especially the four-electrode measurement) allow the monitoring of changes in cell number resulting from different treatments. In addition to demonstrating quantitative variations, longitudinal monitoring of the qualitative, structural changes occurring in cells is naturally receiving significant attention in future research.

## Data Availability

Dataset available on request from the authors.

## Abbreviations

The following abbreviations are used in this manuscript:

AI	Artificial Intelligence
ECIS	Electric Cell-substrate Impedance Sensing
EDL	Electric Double Layer
EIS	Elecrochemical impedance spectroscopy
EIT	Electrical impedance tomography
OoC	Organ-on-Chip
PBS	Phosphate buffered saline
PCB	Printed circuit board
PDMS	Polydimethylsiloxane
PDMS-b-PEO	Poly(dimethylsiloxane-b-ethylene oxide)
TEER	Trans-Epithelial/Endothelial Electrical 45 Resistance
